# Roles of the Tol-Pal system in the Type III secretion system and flagella-mediated virulence in enterohemorrhagic *Escherichia coli*

**DOI:** 10.1038/s41598-020-72412-w

**Published:** 2020-09-23

**Authors:** Hidetada Hirakawa, Kazutomo Suzue, Ayako Takita, Chikako Awazu, Jun Kurushima, Haruyoshi Tomita

**Affiliations:** 1 Department of Bacteriology, Gunma University, Graduate School of Medicine, 3-39-22 Showa-machi Maebashi, Gunma, 371-8511 Japan; 2 Laboratory of Bacterial Drug Resistance, Gunma University, Graduate School of Medicine, 3-39-22 Showa-machi Maebashi, Gunma, 371-8511 Japan; 3 Department of Infectious Diseases and Host Defense, Gunma University, Graduate School of Medicine, 3-39-22 Showa-machi Maebashi, Gunma, 371-8511 Japan

**Keywords:** Antimicrobials, Bacteriology, Microbial genetics, Pathogens, Microbiology, Pathogenesis

## Abstract

The Tol-Pal system is a protein complex that is highly conserved in many gram-negative bacteria. We show here that the Tol-Pal system is associated with the enteric pathogenesis of enterohemorrhagic *E. coli* (EHEC). Deletion of *tolB*, which is required for the Tol-Pal system decreased motility, secretion of the Type III secretion system proteins EspA/B, and the ability of bacteria to adhere to and to form attaching and effacing (A/E) lesions in host cells, but the expression level of LEE genes, including *espA/B* that encode Type III secretion system proteins were not affected. The *Citrobacter rodentium*, *tolB* mutant, that is traditionally used to estimate Type III secretion system associated virulence in mice did not cause lethality in mice while it induced anti-bacterial immunity. We also found that the *pal* mutant, which lacks activity of the Tol-Pal system, exhibited lower motility and EspA/B secretion than the wild-type parent. These combined results indicate that the Tol-Pal system contributes to the virulence of EHEC associated with the Type III secretion system and flagellar activity for infection at enteric sites. This finding provides evidence that the Tol-Pal system may be an effective target for the treatment of infectious diseases caused by pathogenic *E. coli*.

## Introduction

Enterohemorrhagic *Escherichia coli* (EHEC) O157:H7 is a foodborne pathogen that can cause severe diarrhea, hemorrhagic colitis and hemolytic-uremic syndrome (HUS), which can be fatal^1,2^. EHEC produces two major sets of proteins termed Shiga toxins and effector proteins that are responsible for the pathogenicity of this bacterium. The former proteins inhibit protein synthesis within host cells and are closely associated with the development of HUS during infections while the latter proteins are secreted via a protein transport machinery with a needle-like structure termed the Type III secretion system, and trigger the formation of hallmark attaching and effacing (A/E) lesions in host epithelial cells^3–6^. A/E lesions are characterized by the destruction of gut epithelial microvilli, attachment of bacteria to the host cell membrane via the interaction of intimin and its receptor Tir, and formation of a pedestal-like actin-rich structure in the host cell^7^. Effector proteins promote bacteria attachment to host cells, induce rearrangements in the host cell cytoskeleton, and target host cells via translocator proteins, such as EspB^8,9^. A subset of genes that encode Type III secretion system proteins, including effector and translocator proteins, and protein sets of its transport machinery are clustered at a 36 kbp chromosomal pathogenicity island termed the locus of enterocyte effacement (LEE) and transcribed as five major operons (LEE1 to LEE5)^10^.

The Tol-Pal system is a protein complex which traverses the inner membrane, periplasm, and outer membrane in gram-negative bacteria. It was originally characterized in *E. coli*, in which it was shown to be involved in outer membrane maintenance and uptake of colicin and filamentous phage DNA^11,12^. The Tol-Pal system consists of TolA, TolB, TolQ, TolR, and Pal proteins^13,14^. It has been shown that deletion of *tol*-*pal* genes exhibit some phenotypes including increased susceptibility to bile salts and population of filamentous morphology^13,15,16^. Some of *tol*-*pal* genes are also involved in bacterial pathogenesis, such as survival of *Salmonella enterica* serovar Typhimurium within macrophages, cytotoxicity of *Erwinia chrysanthemi* in plant cells, and pustule formation in humans by *Haemophilus ducreyi*^15,17–20^. Previously, we found that mutants in *tol*-*pal* genes of uropathogenic *E. coli* (UPEC) exhibited decreased bacterial internalization in urinary tract cells and impaired motility, which reduced bacterial colonization within the urinary tract of mice^21^. Thus, the Tol-Pal system contributes to the pathogenicity of UPEC in the urinary tract.

We aim to obtain further insights into roles of the Tol-Pal system in pathogenesis of *E. coli*. In this study we focused on characterizing roles of the Tol-Pal system in pathogenesis of EHEC, an enteropathogenic *E. coli* strain that cause infectious diseases at enteric sites, associated with Type III secretion system, Shiga toxin and flagella-mediated motility.

## Results

### Deletion of the *tolB* gene decreases bacterial motility, levels of EspB and EspA, the Type III secretion proteins

**Figure 1:**
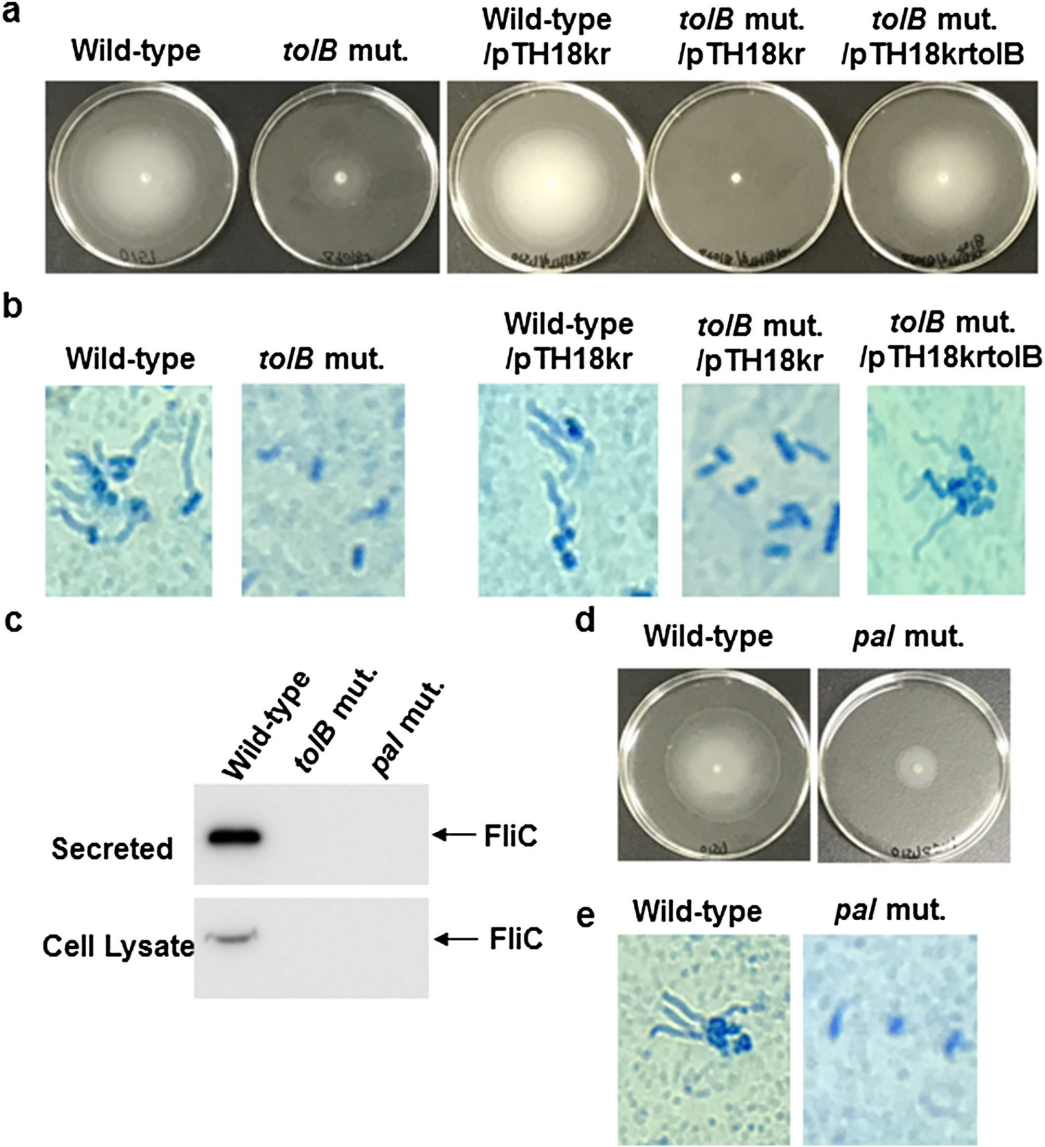
Motilities and flagellar production of the wild-type parent, *tolB* mutant and *pal* mutant, or wild-type parent and *tolB* mutant carrying pTH18kr (empty vector) or pTH18krtolB (*tolB* expression plasmid). (**a**) and (**d**) Bacterial migrations on LB medium containing 0.3% agar were pictured. (**b**) and (**e**) Flagella and bacteria cells stained with Victoria blue/tannic acid were pictured on the microscopy using 100 × objective. (**c**) Cell lysates and secreted proteins from bacteria containing a VSVG-tagged FliC expression plasmid. The proteins including VSVG-tagged FliC were separated by SDS/PAGE, and VSVG-tagged FliC was visualized by western-blotting with VSVG antibody. The full-length blot/gel is presented in Supplementary Fig. 1.

To test if the Tol-Pal system of pathogenic *E. coli* is involved in the pathogenesis at enteric sites, we used EHEC O157:H7 as a typical strain, which causes infectious disease at enteric sites. We constructed an in-frame deletion mutant of the *tolB* gene, which is a member of *tol*-*pal* genes, that lacks the Tol-Pal system. Similar to UPEC, the *tolB* mutant in EHEC exhibited a reduced motility on semi-solid agar compared with the wild-type parent, and the introduction of pTH18krtolB, a heterologous *tolB* expression plasmid, increased its motility up to the level of the wild-type parent (Fig. 1a). We observed that the *tolB* mutant was also less motile than the wild-type parent in broth (see Supplementary Video online). Similar to the *tolB* mutant in UPEC, the *tolB* mutant in EHEC produced defective flagella (Fig. 1b). We also examined the level of flagellin, encoded by *fliC*. Because we were unable to detect FliC even in wild-type EHEC by western-blotting with a commercial FliC antibody, we constructed EHEC strains carrying pTH18krfliC-VSVG, which produces the recombinant VSVG-tagged FliC protein under an innate *fliC* promoter. As predicted, we detected FliC-VSVG in both the cell lysate and secreted fractions from wild-type culture (Fig. 1c) Supplementary Fig. 1). However, FliC-VSVG in those from the *tolB* mutant culture was undetectable. Thus, deletion of *tolB* decreases FliC level, and leads to reduction of flagellar production and motility.

**Figure 2:**
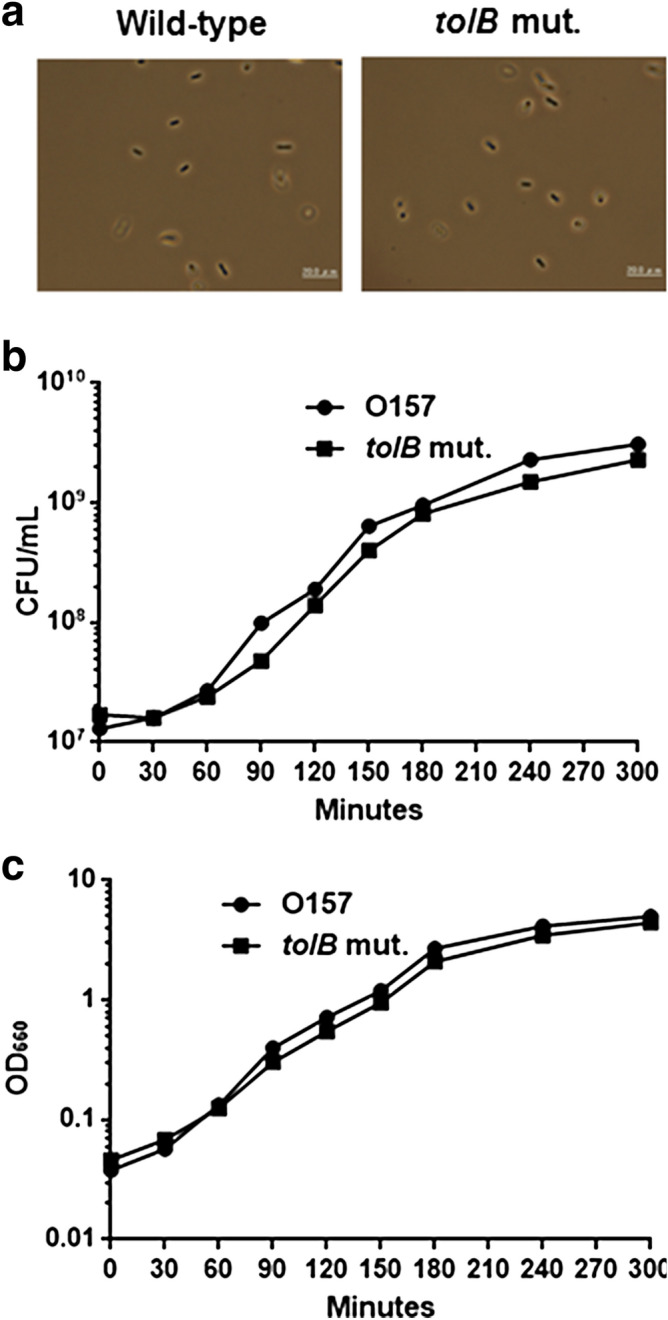
Cell Morphology and growth of the wild-type parent and *tolB* mutant. All strains were grown in LB medium at 37 °C. (**a**) Phase-contrast images of bacteria were pictured on the microscopy using 100 × objective. Bacterial growth were monitored by measuring CFU (**b**) and OD_600_ (**c**).

The *tol*-*pal* mutants in some bacteria including *Vibrio cholerae* and *Erwinia chrysanthemi* exhibited an impaired cell division, and formed extensive filaments during growth^15,16^. However, we did not observe this phenotype in the *tolB* mutant of EHEC. We examined the cell morphology of the *tolB* mutant with the wild-type parent on microscopy. The *tolB* mutant exhibited a rod-shaped morphology similar to the wild-type parent (Fig. 2a). We also compared CFU and OD_600_ values between these strains to assess population of filaments. These values of the *tolB* mutant were moderately lower than those of the wild-type parent, while CFU/OD_600_ values of the *tolB* mutant and the wild-type parent were similar (Fig. 2b,c).

**Figure 3:**
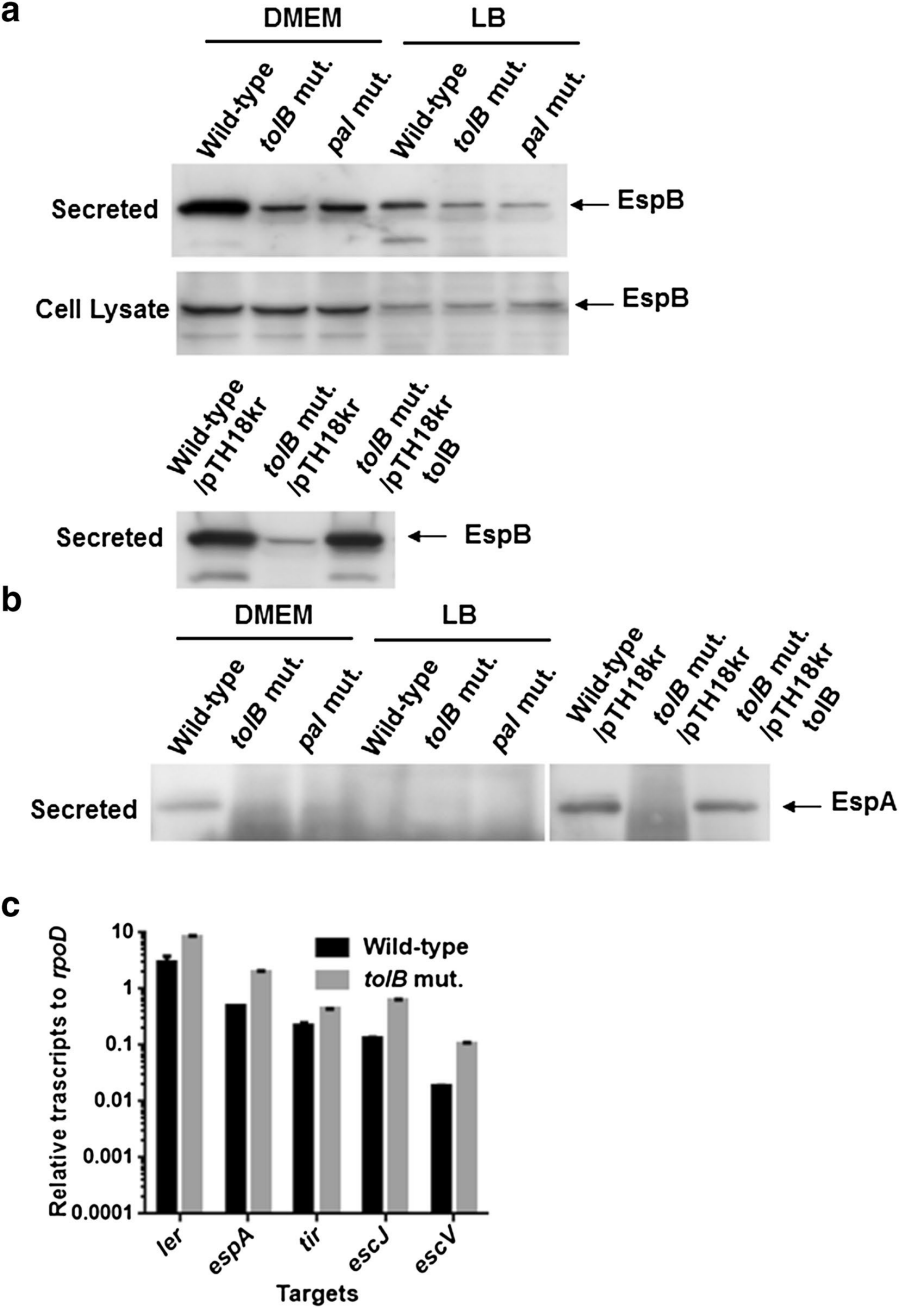
Determination of EspB and EspA levels in culture supernatant and whole cell extract and transcript levels of LEE genes in the wild-type parent, *tolB* mutant and *pal* mutant, or wild-type parent and *tolB* mutant carrying pTH18kr (empty vector) or pTH18krtolB (*tolB* expression plasmid). (**a**, **b**) The wild-type, *tolB* mutant and *pal* mutant were grown in LB medium or DMEM. For complementation test, we grew the wild-type parent and *tolB* mutant carrying pTH18kr or pTH18krtolB in DMEM. The proteins including EspB and EspA were separated by SDS/PAGE, and EspB and EspA were visualized by western-blotting with EspB and EspA antiserums, respectively. Full-length blots/gels are presented in Supplementary Fig. 2 and 3. (**c**) Transcript levels were described as relative values to that of *rpoD* (housekeeping gene). Data plotted are the means of two biological replicates, error bars indicate the ranges.

Type III secretion system proteins are a subset of typical proteins that are involved in the pathogenicity of EHEC. EspB is a member of these proteins, and it is required for the translocation of other effector proteins into host epithelial cells, and this protein also has an effector activity^22–24^. Since most of EspB produced by EHEC is secreted into culture media, we compared the EspB level in culture supernatants from the *tolB* mutant with that from the wild-type parent by western-blotting with an EspB antiserum. The EspB level in the *tolB* mutant was lower than that in the wild-type parent (Fig. 3a) (Supplementary Fig. 2). In contrast, no apparent difference in the levels of intracellular EspB extracted from whole cell fractions in the wild-type parent and *tolB* mutant was observed (Fig. 3a) (Supplementary Fig. 2). We repeated this assay in DMEM. Although LB medium is commonly used for culturing EHEC, Type III secretion system proteins of EHEC are fully activated in expression when grown with DMEM. Levels of both extracellular and intracellular EspB in wild-type EHEC grown with DMEM were higher than those grown with LB. Similar to LB medium, deletion of *tolB* decreased the level of extracellular EspB, but not intracellular EspB (Fig. 3a) (Supplementary Fig. 2). We confirmed that decreased EspB secretion by *tolB* deletion was restored when the pTH18krtolB plasmid for heterologous *tolB* expression was introduced (Fig. 3a) (Supplementary Fig. 2). In addition to EspB, we examined the secretion of EspA, other protein which is secreted via the Type III secretion system. We found that the level of EspA in the *tolB* mutant was also lower than that in the wild-type parent, and the heterologous expression of *tolB* in this mutant elevated EspA up to the wild-type level (Fig. 3b) (Supplementary Fig. 3). We also measured the transcript level of genes that encode Type III secretion system proteins by qPCR. These gene clusters consist of five operons (LEE1–LEE5), and the *espB* gene is transcribed with the *espA* gene in LEE4^10^. As target genes for qPCR analyses, we selected *ler*, *espA*, *tir*, *escJ* and *escV* from each operon. These transcript levels in the *tolB* mutant were moderately higher than those in the wild-type parent, although the reason for this remains unknown (Fig. 3c). These combined results suggest that the *tolB* gene contributes to the secretion, but not the expression of EspB and EspA.

**Figure 4:**
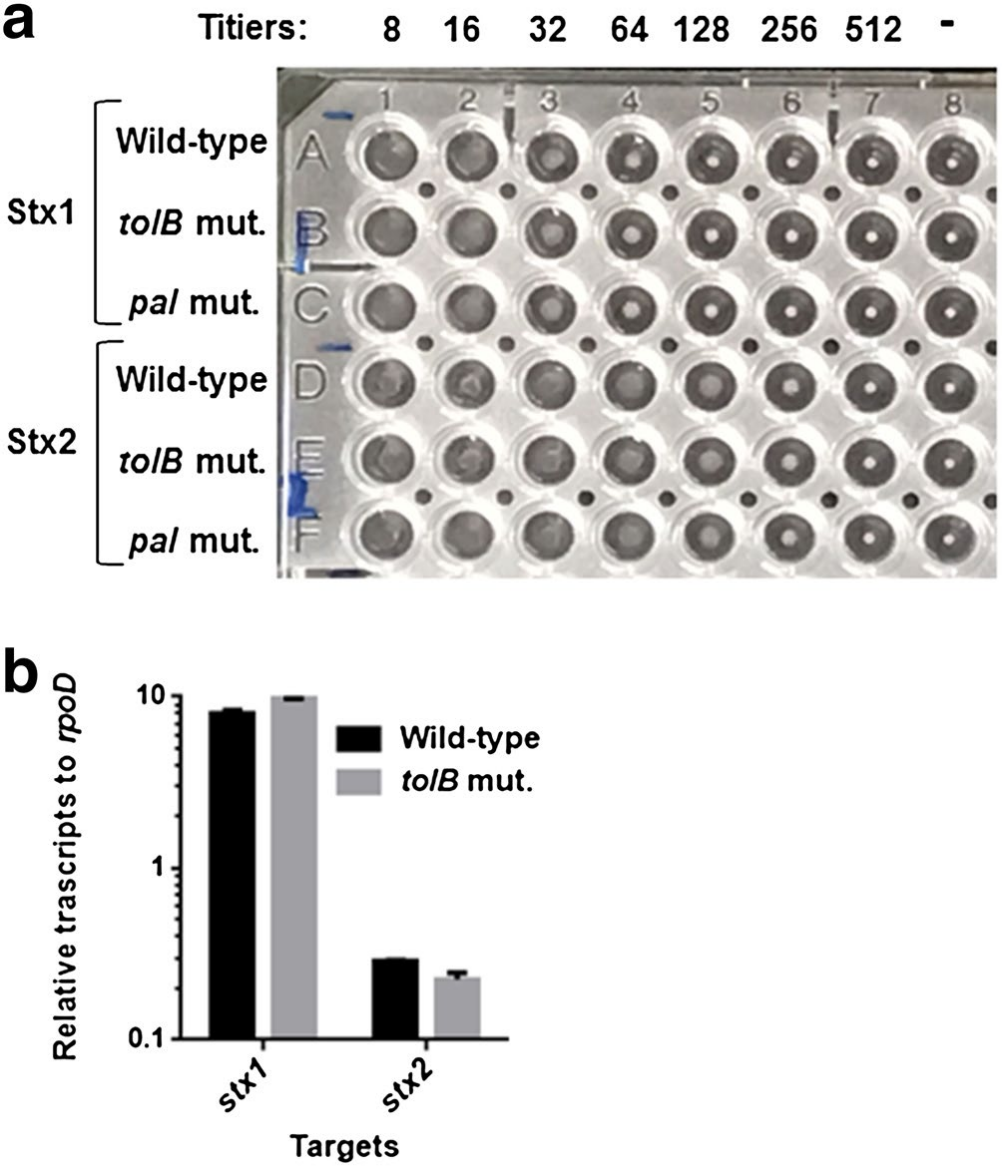
Shiga toxin production of the wild-type parent, *tolB* mutant and *pal* mutant. (**a**) Stx1 and Stx2 latex agglutination titers of culture supernatant from each indicated strain were determined. (**b**) Transcript levels of *stx1* and *stx2*. Transcript levels were described as relative values to that of *rpoD* (housekeeping gene). Data plotted are the means of two biological replicates, error bars indicate the ranges.

We also estimated the activity of Shiga toxins as the other subset of proteins that is required for EHEC virulence, in the wild-type parent and its *tolB* mutant by latex agglutination assays. No significant difference in agglutination titers of both Stx1 and Stx2 was observed between the wild-type and *tolB* mutant (Fig. 4a). We also found that transcript levels of *stx1* and *stx2* in the *tolB* mutant were similar to the wild-type parent (Fig. 4b).

The *tolB* gene product forms protein complexes with Pal, TolA, TolQ, and TolR, proteins, to form the Tol-Pal system. Any members are indispensable for the activity of the Tol-Pal system. We determined whether decreased motility and EspA/B secretion are associated with a defect in the Tol-Pal system. We constructed the *pal* deletion mutant and analyzed motility and EspA/B secretion. The *pal* mutant was less motile than the wild-type parent (Fig. 1d) (see Supplementary Video online) Similar to the *tolB* mutant, the *pal* mutant produced fewer flagella than the wild-type (Fig. 1c,e) (Supplementary Fig. 1). Western-blotting analysis with an EspB antiserum showed a lower level of EspB secretion in the *pal* mutant than that in the wild-type parent while the levels of intracellular EspB in the wild-type parent and the *pal* mutant were similar (Fig. 3a) (Supplementary Fig. 2). We also found that deletion of *pal* decreased the level of EspA secretion, but not Stx1 and Stx2 (Figs. 3b, 4a) (Supplementary Fig. 3). Thus, the *pal* mutant behaves similarly to the *tolB* mutant. These results suggest that the activity of the Tol-Pal system contributes to EHEC motility and virulence.

### The *tolB* mutant exhibits reduced adhesion to epithelial cells, and forms A/E lesions with low efficiency

**Figure 5:**
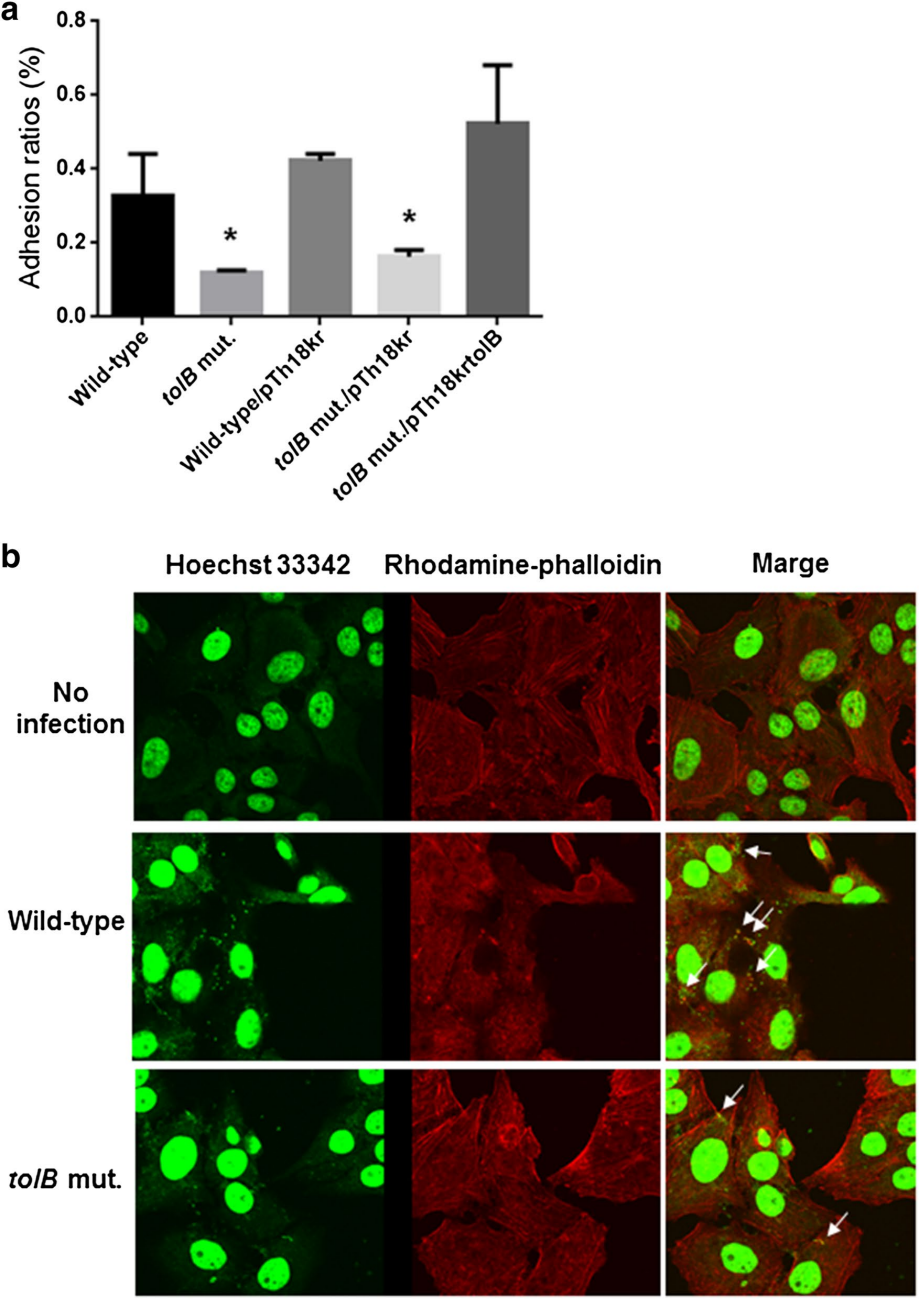
Adhesion to and formation of A/E lesions in HeLa cells for the wild-type parent and *tolB* mutant. (**a**) Y-axis on the graphs shows percent (%) of CFU values of adhered bacteria relative to total bacterial cell numbers. Data plotted are the means; error bars indicate the standard deviations. Asterisks denote significance for values relative to the wild-type control (*P* < 0.05). (**b**) Bacteria and nuclei of HeLa cells stained with Hoechst33342 and actins strained with rhodamine-phalloidin were imaged, respectively, as green and red colors on the microscopy using 100 × objective. Arrows on picture indicate A/E lesions.

EHEC initially adheres to host epithelial cells, and then the bacteria secrete effector and translocator proteins including EspB followed by the establishment of A/E lesions^4,7^. To test if the *tolB* gene also contributes to the initial adhesion process, we compared the ability of the *tolB* mutant to adhere to HeLa cells with that of the wild-type parent. Although EHEC adheres to intestinal epithelial cells in vivo, it also adheres to HeLa cells, and its Type III secretion system proteins, including EspB target the cells in vitro^4,25^. Therefore, HeLa cells are conveniently used to evaluate the in vitro ability of EHEC to adhere to host epithelial cells and determine the activity of the Type III secretion system*.* The *tolB* mutant exhibited an approximately three-fold lower rate of adhesion than the wild-type parent (Fig. 5a). We also observed that the adhesion rate of the *tolB* mutant was comparable to the wild-type level following the introduction of pTH18krtolB (Fig. 5a).

To test whether the *tolB* mutant has a reduced ability to form A/E lesions in epithelial cells compared to the wild-type, HeLa cells were infected with the wild-type parent or the *tolB* mutant, then actin accumulation with bacterial cells in HeLa cells were investigated. We found that the *tolB* mutant produced fewer A/E lesions than the wild-type parent (Fig. 5b). These results indicate that the *tolB* gene is required for optimal adhesion and A/E lesion formation in epithelial cells.

### The *tolB* mutant of *C. rodentium* exhibits low virulence in mice

**Figure 6:**
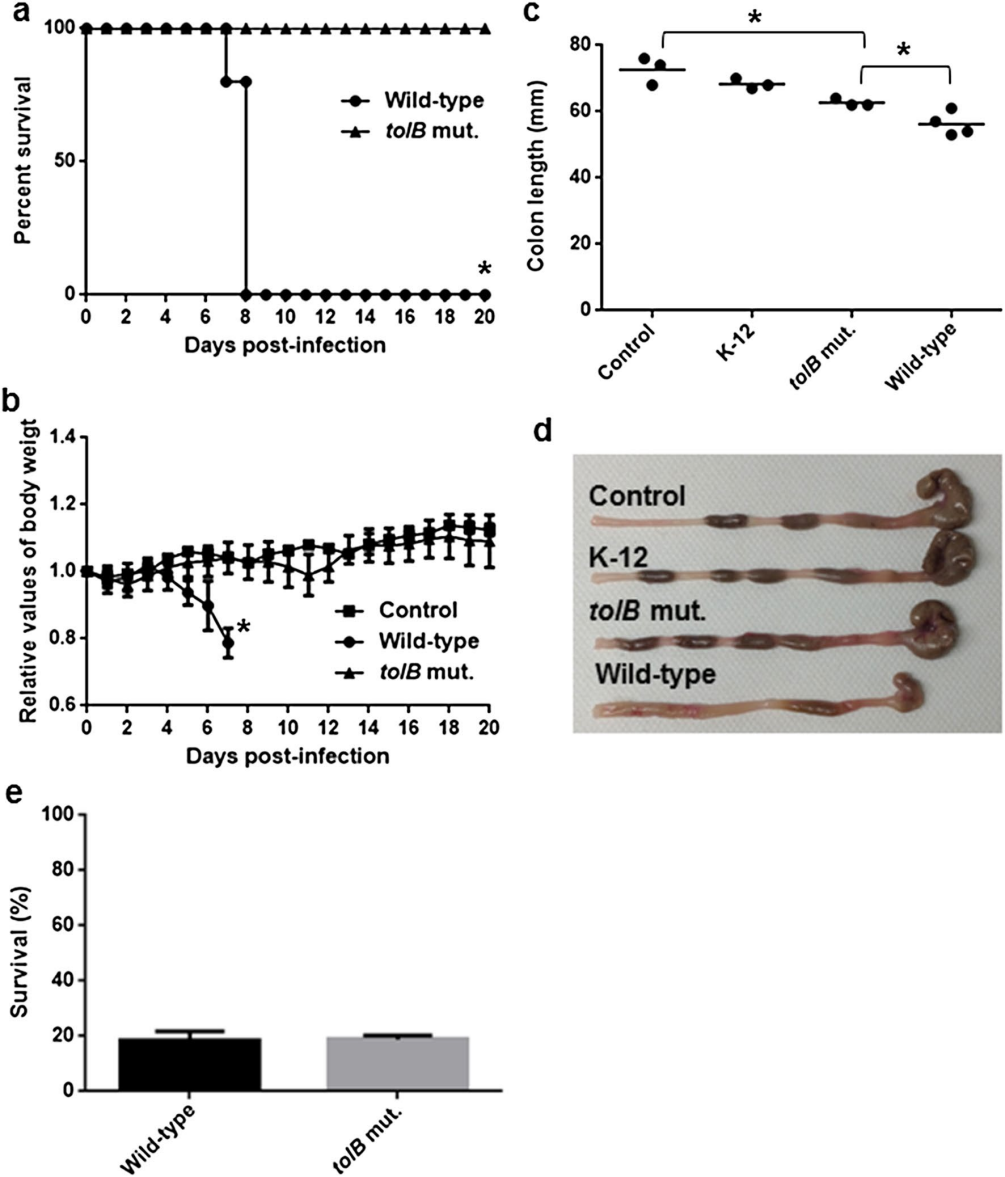
Virulence of the wild-type and *tolB* mutant strains of *C. rodentium* in C3H/HeJ mice. (**a**) Survival of the C3H/HeJ mice infected with the wild-type parent and *tolB* mutant. The mice (N = 5 mice per strain for DBS100 parent and the *tolB* mutant, and N = 2 mice for non-infection control) were daily monitored. (**b**) Change in body weight of the C3H/HeJ mice infected with the wild-type parent and *tolB* mutant. The connecting lines denote the mean and error bars denote range for the data of control mice and standard deviation for the data of mice infected with the parent and *tolB* mutant. Asterisks denote significance for values of survival rate and body weight of mice infected with *tolB* mutant relative to those infected with the parent strain (*P* < 0.05). (**c**, **d**) Length of colons from mice infected with the wild-type parent, *tolB* mutant and non-pathogenic K-12 strain, and control mice (without infection). Colons were isolated at 7 days post-infection. Asterisks denote significance for values of colon length of mice infected with *tolB* mutant relative to those infected with the parent strain, and control mice (*P* < 0.05). (**e**) Survival of the wild-type parent and *tolB* mutant after challenged at an acidic condition (pH3.0). Y-axis on the graphs shows percent (%) of CFU values of cells after incubation in the acidified LB medium relative to CFU values of cells after incubation in the regular LB medium. Data plotted are the means; error bars indicate the standard deviations.

To characterize the role of the *tolB* gene in the Type III secretion system-associated with EHEC pathogenesis in vivo, we used a murine intestinal infection model with *C. rodentium*. Since EHEC is a human-specific pathogen, it does not cause typical symptoms of the disease in mice that reflect those observed in human infections. *C. rodentium* is a natural pathogen of mice, and it carries LEE genes but not genes that encode Shiga toxins^26^. For this reason, *C. rodentium* is frequently used to evaluate the Type III secretion system associated virulence in mice. We constructed the *tolB* deletion mutant from the *C. rodentium* DBS100 strain that is highly virulent in C3H/HeJ mice. When the mice were administrated with the wild-type parent, all mice died within 8 days following a marked decrease in body weight on the previous day (Fig. 6a,b). In contrast, the mice infected with the *tolB* mutant survived for at least 21 days. A moderate decline in body weight was observed in these mice until the twelfth day post-infection, but their body weight increased thereafter, which implies the recovery from infection. We also characterized intestinal symptoms in mice at 7 days post-infection. The colons of mice infected with wild-type *C. rodentium* exhibited a significantly shorter in the length than those with MG1655, the non-pathogenic K-12 strain or mice of non-infection group (Fig. 6c). Diarrhea-like stool inside of the colon and some swelling were observed in mice infected with wild-type *C. rodentium*, suggesting that the wild-type *C. rodentium* causes inflammation of the colons (Fig. 6d). On the other hand, mice infected the *tolB* mutant exhibited relatively short colons compared to the control mice, but still significantly longer than mice infected with the wild-type (Fig. 6c). Similar to the control mice, mice infected the *tolB* mutant exhibited no apparent swelling on the colon and also showed normal stool inside of the colon (Fig. 6d). These combined results indicate that the *tolB* gene of *C. rodentium* is necessary for optimal virulence in mice infected at the enteric site. However, the *tolB* mutant may be more susceptible to acid compared with the wild-type parent, which may attribute the low virulence of this mutant to a low ability of gastric transit under acidic conditions after oral infection. To verify this possibility, we tested the bacterial survival of the DBS100 parent and *tolB* mutant after incubation in an acidified medium (pH 3.0). No significant difference in the survival rates between the parent and *tolB* mutant was observed (Fig. 6e). Therefore, the low virulence of the *tolB* mutant is not solely due to a high susceptibility to acid. It is known that *tol*-*pal* mutants are highly susceptible to bile salts^13,15^, therefore deletion of *tol*-*pal* genes may reduce fitness cost in intestinal tracts, which may explain low virulence of the *tolB* mutant. We found that the *tolB* mutant of *C. rodentium* DBS100 exhibited lower MICs of bile salts, such as sodium cholate and sodium deoxycholate than the wild-type parent (Table 1).

**Table 1:** Bile salt MICs for *C. rodentium* and EHEC, and its *tolB* mutants.

Strain	MICs (mg/L) of	
	Sodium cholate	Sodium deoxycholate
O157	> 51,200	> 51,200
O157ΔtolB	12,800	1,600
DBS100	51,200	12,800
DBS100ΔtolB	12,800	1,600

### Anti-bacterial immunity is elicited upon *tolB* mutant infection

**Figure 7:**
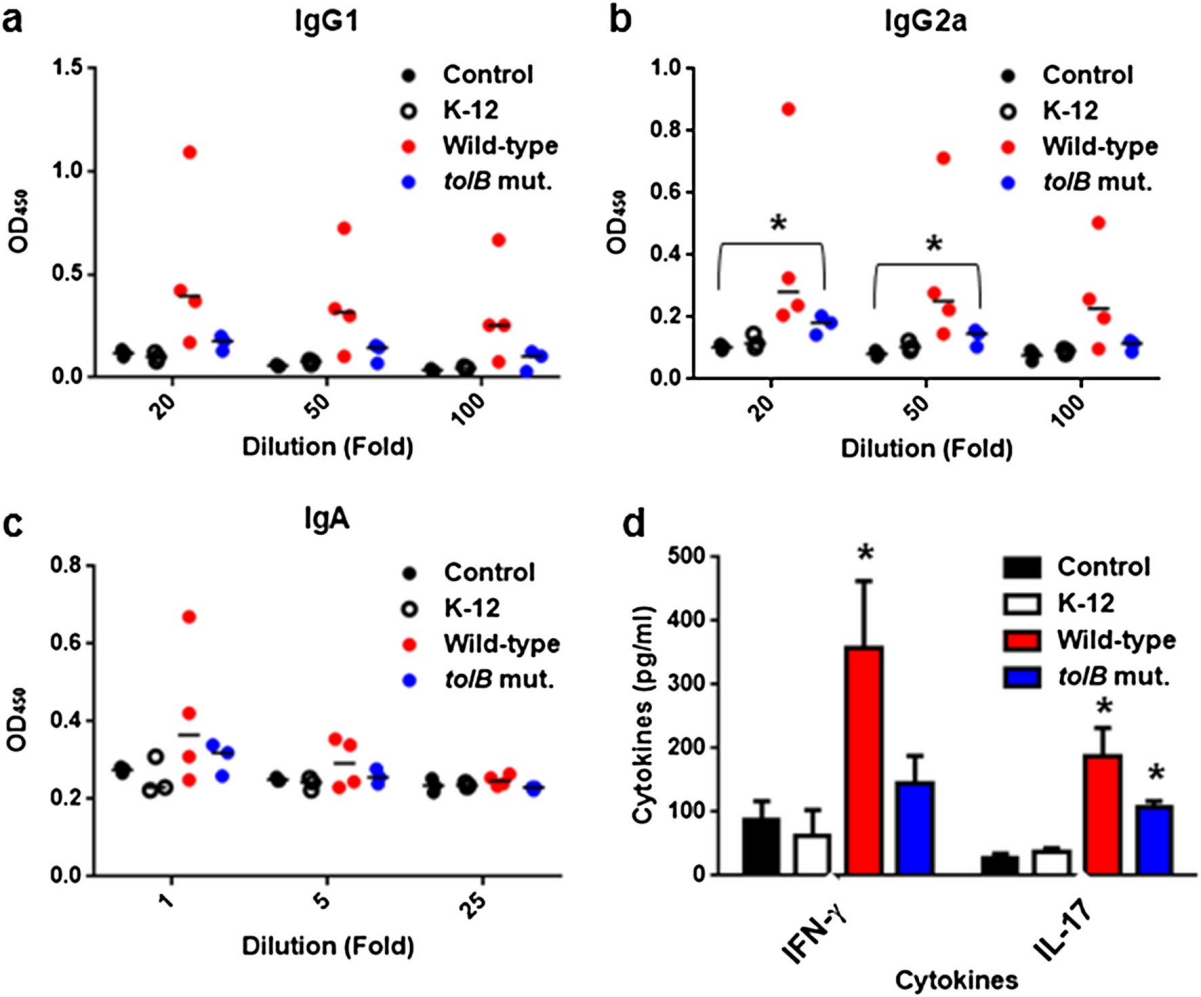
Immune response in mice infected with the wild-type parent, *tolB* mutant and non-pathogenic K-12 strain, and control mice (without infection). (**a**–**c**) Levels of IgG1 and IgG2a from serum and IgA from feces in mice at 7 days post-infection. Each data point represents a sample from an individual mouse. Horizontal bars indicate the mean values. (**d**) Levels of IFN-γ and IL-17 in spleen cells of mice, re-stimulated with an extract from *C. rodentium* DBS100. Data plotted are the means; error bars indicate the standard deviations. Asterisks denote significance for levels of antibodies and cytokines of mice infected with the wild-type or *tolB* mutant relative to those of control mice (*P* < 0.05).

To investigate an ability of immune response against high virulent wild-type *C. rodentium*, in mice infected with the *tolB* mutant, we tested the production of anti-bacterial antibodies and cytokines elicited by activation of T cells. Initially, we found that mice infected with the *tolB* mutant produced a significant amount of anti-bacterial IgG2a, but not IgG1 and IgA antibodies at 7 days post-infection compared to control mice and mice infected with the non-pathogenic K12 strain (Fig. 7a–c). To test an ability of T cell response, we determined levels of Th1 cytokine IFN-γ and Th17 cytokine IL-17 produced from spleen cells after in vitro re-stimulation with an extract from *C. rodentium* DBS100. We observed that a significant amount of IL-17 production was elicited in spleen cells from mice infected with the *tolB* mutant (Fig. 7d). These combined results indicate that *tolB* mutant bacteria have the ability to elicit the immune response against wild-type virulent strain.

## Discussion

The Tol-Pal system was first characterized in *E. coli*, and it consists of two protein complexes formed by TolA, TolB, TolQ, TolR and Pal^13,14^. The subset of *tol*-*pal* genes that encode these proteins is also found in many species of other gram-negative bacteria. It has been shown that these Tol-Pal proteins are involved in the pathogenesis^15,17–20^. In this study, we suggest that the Tol-Pal system contributes to EHEC virulence associated with the Type III secretion system and flagellum because the *tolB* and *pal* deletion mutants that lack the activity of the Tol-Pal system exhibited decreased functional flagella production, secretion of the Type III secretion system proteins, EspA and EspB required for the formation of A/E lesions, and ability to adhere to and to form A/E lesions in host epithelial cells. We also showed that the *tolB* mutant of *C. rodentium* did not cause lethality in mice, whereas all mice infected with the parent strain died within 8 days post-infection. In addition, unlike mice infected with the wild-type *C. rodentium*, mice infected with the *tolB* mutant did not exhibit symptoms of inflammation and diarrhea-like stool in the colon. This supports the results of in vitro experiments using EHEC. *C. rodentium* is traditionally used to evaluate virulence associated with the Type III secretion system in the intestine of mice. The pathogenesis of this bacterium reflects that of EHEC associated with the Type III secretion system in mice because *C. rodentium* produces a subset of Type III secretion system proteins that share high sequence similarity with that in EHEC, but it dose not produce Shiga toxins^26^. The *tolB* mutant exhibited a higher susceptible to bile salts than the wild-type parent. This may be also involved in attenuated virulence of the *tolB* mutant in mice.

Results of antibody and T cell response suggest that the *tolB* mutant has the ability to elicit the immune response, including activation of Th17 cells, against wild-type virulent strain although this induction is still moderate. Activation of Th17 cells is proposed to be important for protection from some enteric-pathogens including *C. rodentium* for mice^27^, which implies that the *tolB* mutant give a clue to develop the low virulent live vaccine against EHEC without affecting the potential of eliciting the protective immunity.

Type III secretion system proteins are the major proteins responsible for virulence in the human intestine because these proteins enable EHEC to induce A/E lesions in enteric epithelial cells, and the induction of A/E lesions leads to severe haemorrhagic diarrhea^4^. During infection, EHEC initially adheres to intestinal epithelial cells, on which it produces a subset of Type III secretion system proteins, and then injects these effector proteins into host cells via transport machinery^28^. Therefore, bacterial adhesion to host cells is an initial key step that enables EHEC to induce virulence associated with the Type III secretion system followed by the establishment of A/E lesions in host intestinal cells. When EHEC adheres to cells, it uses proteins localized in the outer membrane including fimbria, adhesin proteins and flagella^29,30^. The *tolB* and *pal* mutants produced defective flagella in addition to decreased EspB secretion. Expression of the flagellum and Type III secretion system proteins is inversely regulated because the expression of *fliC* which encodes flagellin is repressed when the production of Type III secretion system proteins is induced at later stages after the initial attachment via flagellin^31^. Our results suggest that the activity of the Tol-Pal system in EHEC may regulate both the initial attachment and induction of A/E lesions in host epithelial cells.

The *tol*-*pal* mutants that lack activity of the Tol-Pal system exhibited defects in some components localized on the outer-membrane, such as lipopolysaccharide and OmpA protein^32^. In this study, we also found that secretion of EspA/B, the translocator proteins secreted via the Type III secretion system, was decreased following the deletion of *tolB* and *pal* genes. However, the intracellular EspA/B level and transcript levels of some genes in LEE operons, including *esp*A and *espB* were not decreased. We speculate that the activity of the Tol-Pal system may also be involved in the assembly of the transport protein complex for the Type III secretion system, and thus, the secretion ability of effector and translocator proteins including EspA/B in *tolB* and *pal* mutants may be defective, which leads to the reduced secretion of these proteins compared with the wild-type parent. In the transport protein complex, EscC is a major protein required to form the “outer ring” embedded in the outer membrane, and it is associated with both EscD, an inner membrane protein and the EscF needle structure protein^33–36^. The disturbance in the outer membrane by deletion of *tol*-*pal* genes may impair the precise localization and stabilization of the outer ring, which presumably leads to disassembly of the transport protein complex. However, some proteins localized in the outer membrane, such as fimbrial protein complexes, remain functional in the *tol*-*pal* mutants despite the absence of activity of the Tol-Pal system^21^. It would be interesting to gain insight into which proteins are affected by the Tol-Pal system in addition to Type III transport proteins and how these proteins are associated with it.

In addition to the results of our previous study with UPEC, we showed here that the Tol-Pal system also contributes to the virulence of EHEC, which causes an infectious disease at enteric sites. This finding may enable us to make the idea that attenuates bacterial virulence by targeting the Tol-Pal system as an option for chemotherapies to treat infections caused by pathogenic *E. coli*.

## Materials and methods

### Bacterial strains, host cells and culture conditions

The bacterial strains and plasmids used in this study are listed in Table 2. Bacteria were grown in Luria–Bertani (LB) medium unless otherwise indicated. The optical density at 600 nm (OD_600_) was measured as an indicator of cell growth. The antibiotics chloramphenicol (45 μg/mL) and kanamycin (50 μg/mL) were added to the growth media for marker selection and maintaining plasmids. HeLa cells, the cervical cancer cells were cultured in Dulbecco’s Modified Eagle Medium containing 10% HyClone FetalClone III serum (HyClone Laboratories, Inc., Logan, UT, United States) at 37 °C and in an atmosphere of 5% CO_2_.

**Table 2:** Strains and plasmids used in this study.

Strain or plasmid	Relevant genotype/phenotype	References
**Strains**		
O157	EHEC O157:H7 [RIMD0509952]	^38^
O157ΔtolB	*tolB* mutant from O157	This work
O157Δpal	*pal* mutant from O157	This work
DBS100	*Citrobacter rodentium* (ATCC 51459)	^39^
DBS100ΔtolB	*tolB* mutant from DBS100	This work
MG1655	*E. coli* non-pathogenic K-12 strain	^40^
**Plasmids**		
pKO3	Temperature sensitive vector for gene targetting, *sacB*, Cm^R^	^37^
pTH18kr	low copy number plasmid; Km^R^	^41^
pTH18krtolB	*tolB* expression plasmid; Km^R^	this work
pTH18krfliC-VSVG	C-terminal VSVG-tagged FliC expression plasmid; Km^R^	this work

Cm^R^, chloramphenicol resistance; Km^R^, kanamycin resistance.

### Cloning and mutant construction

In-frame gene deletions were produced by sequence overlap extension polymerase chain reaction (PCR), as described previously^37^, using the primer pairs delta1/delta2 and delta3/delta4 (Table 3). The upstream flanking DNA comprised 450 bp and the first two amino acid codons of *tolB,* and the first three amino acid codons of *pal*. The downstream flanking DNA included, the last amino acid codon (CTG) of *tolB* in EHEC, the last six amino acid codons of *tolB* in *C. rodentim*, the last five amino acid codons of *pal*, a stop codon, and 450 bp of DNA. These deletion constructs were ligated into the temperature-sensitive plasmid pKO3^37^ and introduced into EHEC or the *C. rodentium* strain. We selected sucrose-resistant/chloramphenicol-sensitive colonies at 30 °C. We also constructed pTH18krtolB. The *tolB* gene and its 200 bp upstream were cloned into the low-copy number plasmid pTH18kr. To construct a C-terminal vesicular stomatitis virus glycoprotein (VSVG)-tagged FliC expression plasmid, pTH18krfliC-VSVG, the DNA containing the *fliC* coding region and its 300-bp upstream region was amplified with pTHfliC-F and pTHfliC-VSVG-R primers, and ligated into HindIII and BamHI sites in pTH18kr. This DNA fragment contains a cis-regulatory element for *fliC* gene expression. In addition, although the pTH18kr vector has a *lac* promoter sequence upstream of the HindIII and BamHI sites, the introduced *fliC* gene is oriented in a reverse direction to the *lac* promoter. Therefore, we expected that the resulting bacteria construct would produce FliC as a C-terminal VSVG-tagged protein from its native promoter. All constructs were confirmed by DNA sequencing.

**Table 3:** Primers used for plasmid construction.

Primer	DNA sequence (5′ – 3′)	Use
tolB-EHECdelta1	gcggcggccgcgtgagctaagctctggtaag	*tolB* mutant construction for O157
tolB -EHECdelta2	ctattcaattaattattatcacagcttcatcatatctcccttatc	*tolB* mutant construction for O157
tolB -EHECdelta3	gataagggagatatgatgaagctgtgataataattaattgaatag	*tolB* mutant construction for O157
tolB -EHECdelta4	gcggtcgacgtactgcaggtttttctttac	*tolB* mutant construction for O157
pal-EHECdelta1	Gcggcggccgccgacctggttcccggacag	*pal* mutant construction for O157
pal-EHECdelta2	ttctcttagtaaaccagtaccgccagttgcatttcaatgattcc	*pal* mutant construction for O157
pal-EHECdelta3	aaggaatcattgaaatgcaactggcggtactggtttactaagag	*pal* mutant construction for O157
pal-EHECdelta4	gcggtcgacgatttatcctgcaccagcgc	*pal* mutant construction for O157
tolB-CRdelta1	gcggcggccgcagctccggtaagaatgcgc	*tolB* mutant construction for DBS100
tolB-CRdelta2	tatcacagatacggcgaccaggccttcatcatatctcccttatc	*tolB* mutant construction for DBS100
tolB-CRdelta3	cggataagggagatatgatgaaggcctggtcgccgtatctgtg	*tolB* mutant construction for DBS100
tolB-CRdelta4	gcggtcgacgtactgcaggtttttctttac	*tolB* mutant construction for DBS100
pTH-tolB-F	gcggtcgacatcccgaaaccaccaagccag	pTH18krtolB construction
pTH-tolB-R	gcgaagctttcacagatacggcgaccagg	pTH18krtolB construction
pTH-fliC-F	gcgaagcttcctgacccgactcccagcg	pTH18krfliC-VSVG construction
pTH-fliC-VSVG-R	gcgggatccttattttcctaatctattcatttcaatatctgtataaccctgcagcagagacag	pTH18krfliC-VSVG construction

### EspA antiserum

EspA antiserum was produced in rabbits by Sigma-Aldrich Co. LLC (St. Louis, MO, United States). A peptide synthesized from positions 92 to 110 of EspA was used as an antigen.

### Motility assays

LB medium containing 0.3% agar was spotted with 2 μL of bacteria grown for 18 h at 37 °C in LB medium. Bacterial motility was evaluated by measuring the motility diameters after 8 h at 37 °C under an atmosphere of 5% CO_2_. We also monitored bacterial motility in liquid cultures. Bacteria were grown to the late logarithmic growth phase in LB medium, and phase-contrast images of bacterial motility were recorded on a Carl Zeiss Axiovert 200 microscope using a 100 × objective and captured with an Olympus DP71camera.

### Flagellum staining

Bacteria were cultured for 24 h at 30 °C in Heart Infusion medium containing 1.5% agar. Flagella were stained with Victoria blue/tannic acid solution as described previously^21^.

### Western blotting

EHEC strains were grown at 37 °C with shaking to the early stationary phase, and separated by centrifugation and filtration. Secreted proteins were precipitated from the supernatants with 10% trichloroacetic acid (TCA) and dissolved in Laemmli sample buffer (Bio-Rad Laboratories, Hercules, CA, United States). Intracellular proteins were resuspended in 50 mM phosphate buffer containing 8 M urea and then lysed by sonication. Cell lysates (12 μg) and secreted proteins were separated on a 12.5% acrylamide for EspA and EspB detection or 10% acrylamide for VSVG-tagged FliC detection Tris–glycine SDS/PAGE gel. The gel was electroblotted onto a polyvinylidene fluoride (PVDF) membrane (Bio-Rad Laboratories, Hercules, CA, United States). EspA, EspB and VSVG-tagged FliC were detected with EspA antiserum, EspB antiserum^42^, VSVG antibody (Sigma-Aldrich Co. LLC., St. Louis, MO, United States), an anti-rabbit horseradish peroxidase-conjugated immunoglobulin G (IgG) secondary antibody (Sigma-Aldrich Co. LLC., St. Louis, MO, United States) and a SuperSignal West Pico Kit (Thermo Fisher Scientific, Waltham, MA, United States). These protein bands were visualized on a LAS-4000 Luminescent Image Analyzer (GE Healthcare Japan, Tokyo). All gel/blot images were acquired and processed by using Image Quant LAS 4000 software (GE Healthcare Japan, Tokyo, Japan).

### Shiga toxins assay

To test the activity of Shiga toxins (Stx1 and Stx2), we used latex agglutination reagents (Denka Seiken Co. Ltd, Tokyo, Japan). EHEC strains were grown at 37 °C with shaking to the early stationary phase in Muller–Hinton medium, and separated by centrifugation and filtration. These culture supernatants were serially diluted in 96-well round bottom plates containing phosphate-buffered saline (PBS) and an equal volume of the latex suspension sensitized with Stx1 or Stx2 antibody was then added. After incubation for 14 h at 4 °C, titers were determined. The titers are presented as the reciprocal of the dilution of the last well before agglutinations were observed.

### RNA extraction and quantitative real-time PCR analyses

Bacteria were grown to the late logarithmic growth phase at 37 °C in LB medium. Total RNA extraction and cDNA synthesis were performed using a SV Total-RNA Isolation System (Promega Corp., Madison, WI, United States) and ReverTra Ace qPCR RT Manster Mix (TOYOBO Co. Ltd, Osaka, Japan), respectively, following the manufacturer’s instructions. Real-time PCR were carried out as described previously^21^. Primers were listed in Table 4.

**Table 4:** Primers used for real-time PCR analyses.

Primer	DNA sequence (5′–3′)	Use
rrsA-qPCR-F	cggtggagcatgtggtttaa	Quantitative real-time PCR
rrsA-qPCR-R	gaaaacttccgtggatgtcaaga	Quantitative real-time PCR
rpoD-qPCR-F	caagccgtggtcggaaaa	Quantitative real-time PCR
rpoD-qPCR-R	gggcgcgatgcacttct	Quantitative real-time PCR
ler-qPCR-F	cgaccaggtctgcccttct	Quantitative real-time PCR
ler-qPCR-R	tcgctcgccggaactc	Quantitative real-time PCR
espA-qPCR-F	ccgttgtcaggttattcgcttt	Quantitative real-time PCR
espA-qPCR-R	tgatttaagcgctggtgatctg	Quantitative real-time PCR
tir-qPCR-F	tttttgcgcctgagcattatt	Quantitative real-time PCR
tir-qPCR-R	gctaaagcagcaggcgaaga	Quantitative real-time PCR
escJ-qPCR-F	aaagaagctaatcagatgcaagca	Quantitative real-time PCR
escJ-qPCR-R	tccacttttgtccatttctttgg	Quantitative real-time PCR
escV-qPCR-F	cgcctgcggtgacagaa	Quantitative real-time PCR
escV-qPCR-R	ttgtctgtcggtgatgctttg	Quantitative real-time PCR
stx1-qPCR-F	tcgcgagttgccagaatg	Quantitative real-time PCR
stx1-qPCR-R	ttccatctgccggacacat	Quantitative real-time PCR
stx2-qPCR-F	ttcgcgccgtgaatgaa	Quantitative real-time PCR
stx2-qPCR-R	caggcctgtcgccagttatc	Quantitative real-time PCR

### Bacterial adhesion to host epithelial cells

Bacterial adhesion to HeLa cells were assessed as s described previously with slight modifications^21^. Bacteria were inoculated into HeLa cells in 24-well plates. After incubation for 4 h, the total number of bacteria was determined from a first set of duplicate wells. The number of adhered bacteria was determined by counting the number of bacteria present in a second set of wells after washing five times with PBS + (PBS containing 0.5 mM MgCl_2_ and 1 mM CaCl_2_). Numbers of adhered bacterial cells are represented as relative colony forming units (CFUs) by their ratios (%) to total cell CFUs.

### Assessment of A/E lesion formation

Bacteria were inoculated with an MOI of 100 bacteria per host cell into cultured HeLa cells on glass coverslips in a 6-well plate and incubated at 37 °C for 4 h, then the medium was replaced by a fresh medium. The culture was continued for another 2 h. The coverslips were washed with PBS+. Rhodamine phalloidin (Life Technologies, Carlsbad, CA, United States) was used to visualize actin, and Hoechst33342 (Dojindo Laboratories, Tokyo, Japan) was used to stain bacteria and HeLa nuclei. Fluorescent images were acquired in the DAPI and rhodamine phalloidin laser units on an Olympus FV1200 IX81 microscope using a 100 × objective and captured with a CCD camera.

### *C. rodentium* infections in mice

Four week old female C3H/HeJ mice were obtained from CLEA Japan (Tokyo, Japan). *C. rodentium* DBS100 or its *tolB* mutant was grown overnight in LB medium with shaking (180 rpm) at 37 °C. The bacterial cells were harvested, and resuspended in fresh LB medium at a concentration of 1 × 10^9^ CFU/mL, and 200 μL of the bacterial suspension (2 × 10^8^ CFU) was orally administrated. As a control group, 200 μL of bacteria-free LB broth and/or culture resuspension from MG1655, a non-pathogenic K-12 strain of *E. coli*, were inoculated into mice. To measure survival rates and body weight of mice, the mice were monitored daily for 21 days. To characterize intestinal symptoms, mice were euthanized 7 days post-infection and the colons were aseptically removed.

### Acid survival assays

Bacteria were grown overnight in LB medium with shaking (180 rpm) at 37 °C. A 10 μL bacterial suspension (2 × 10^8^ CFU) from the overnight culture was transferred to 990 μL of acidified LB medium (pH 3.0, adjusted with HCl) or regular LB medium (pH 7.2) and incubated for 1 h. Bacterial CFUs were determined by plating serial dilutions on LB agar, and then percentage survival was calculated as the number of CFUs for cells after incubation in the acidified LB medium relative to the number of CFUs for cells after incubation in the regular LB medium.

### Bile salts susceptibility assays

To test susceptibility of bacteria to bile salts, we determined minimum inhibitory concentrations (MICs) of sodium deoxycholate and sodium cholate, that are major components of bile salts, by using a serial agar dilution method. Five microliters of 100-fold-diluted overnight cultures (~ 50,000 cells) was inoculated onto a LB agar plate containing sodium deoxycholate or sodium cholate and incubated for 18 h at 37 °C. The MICs were determined as the lowest concentration at which growth was inhibited.

### Preparation of *C. rodentium* antibodies

*C. rodentium* DBS100 was grown overnight in LB medium with shaking (180 rpm) at 37 °C. Bacterial cells were harvested, then lysed by freeze–thaw and sonication. Bacterial extracts were prepared in PBS with a concentration of 50 μg/mL to detect serum IgGs and fecal IgA by ELISA according to manufacturer’s protocol (Bethyl Laboratories, Montgomery, TX, United States)^43^. To detect fecal IgA, fecal extracts were prepared from fresh feces^43^.

### In vitro anti-bacterial T cell response

Spleen cells were isolated from mice infected with bacteria and control mice, and 1 × 10^6^ cells were re-suspended in 0.2 mL of RPMI1640 medium and seeded in each well of 96-well culture plates^44^. Cells were stimulated with bacterial antigens from 1 × 10^6^ cells of *C. rodentium* DBS100 killed by freeze–thaw, and incubated for 48 h. IFN-γ and IL-17 in culture supernatants were assayed by using ELISA kits in accordance with manufacturer’s protocols (eBioscience product from Thermo Fisher Scientific, Waltham, MA, United States).

### Approval for experiments

All experimental protocols were approved by the Gunma University Gene Recombination Experiment Safety Committee for all gene recombination and bacteria culture studies (The approval number: 16-002) and by the Committee of Experimental Animal Research of Gunma University for all animal studies (The approval number: 19-094). All experiments were performed in accordance with these committee’s guidelines and regulations.

## Supplementary information

Supplementary Figures.

Supplementary Video a.

Supplementary Video b.

Supplementary Video c.

## Data Availability

All data generated or analysed during this study are included in this published article.
