# Supplementary material for: Roles of the Tol-Pal system in the Type III secretion system and flagella-mediated virulence in enterohemorrhagic Escherichia coli
Source: Sci Rep. 2020 Sep 23;10:15173. doi: 10.1038/s41598-020-72412-w (PMC7511404; doi:10.1038/s41598-020-72412-w)

**Roles of the Tol-Pal system in the Type III secretion system and flagella-mediated virulence in enterohemorrhagic *Escherichia coli***

Hidetada Hirakawa^1^,

Kazutomo Suzue^3^

Ayako Takita^1^

Chikako Awazu^3^

Jun Kurushima^1^

Haruyoshi Tomita^1,2^

^1^Department of Bacteriology, ^2^Laboratory of Bacterial Drug Resistance and ^3^Department of Infectious diseases and host defense, Gunma University, Graduate School of Medicine, 3-39-22 Showa-machi Maebashi, Gunma 371-8511, Japan

**Supplementary figure legends**

**Supplementary figure 1** Cell lysates and secreted proteins from the wild-type, *tolB* mutant and *pal* mutant containing a VSVG-tagged FliC expression plasmid. Bacteria were grown in LB medium or DMEM. The proteins including VSVG-tagged FliC were separated by SDS/PAGE, and VSVG-tagged FliC was visualized by western-blotting with VSVG antibody. Locations of molecular mass standards (in kilodaltons) are shown on the left (Super Signal Enhanced Molecular Weight Protein Ladder; Thermo Fisher Scientific, Waltham, MA, United States). The peptide size of VSVG-tagged FliC is approximately speculated to be 61 kilodaltons.

**Supplementary figure 2** Cell lysates and secreted proteins from the wild-type, *tolB* mutant and *pal* mutant, or wild-type parent and *tolB* mutant carrying pTH18kr (empty vector) or pTH18krtolB (*tolB* expression plasmid). The wild-type, *tolB* mutant and *pal* mutant were grown in LB medium or DMEM. For complementation test, we grew the wild-type parent and *tolB* mutant carrying pTH18kr or pTH18krtolB in DMEM. The proteins including EspB were separated by SDS/PAGE, and EspB was visualized by western-blotting with EspB antiserum. Locations of molecular mass standards (in kilodaltons) are shown on the left (Super Signal Enhanced Molecular Weight Protein Ladder; Thermo Fisher Scientific, Waltham, MA, United States). The peptide size of EspB is approximately speculated to be 33 kilodaltons.

**Supplementary figure 3** Cell lysates and secreted proteins from the wild-type, *tolB*, and *pal* mutant, or wild-type parent and *tolB* mutant carrying pTH18kr (empty vector) or pTH18krtolB (*tolB* expression plasmid). The wild-type, *tolB* mutant and *pal* mutant were grown in LB medium or DMEM. For complementation test, we grew the wild-type parent and *tolB* mutant carrying pTH18kr or pTH18krtolB in DMEM. The proteins including EspA were separated by SDS/PAGE, and EspA was visualized by western-blotting with EspA antiserum. Locations of molecular mass standards (in kilodaltons) are shown on the left (Super Signal Enhanced Molecular Weight Protein Ladder; Thermo Fisher Scientific, Waltham, MA, United States). EspA overexpression on the right lane indicates the cell lysate from the *E. coli* Rosetta strain containing the isopropyl-β-D-thiogalactopyranoside (IPTG)-inducible *espA* overexpression plasmid (pTrc99AespA). The peptide size of EspA is approximately speculated to be 21 kilodaltons.

**Supplementary video** Migrations of the wild-type parent, *tolB* mutant and *pal* mutant. The wild-type parent (a), *tolB* mutant (b) and *pal* mutant (c) were grown in LB medium, then 5 μl of bacterial cultures were placed onto dish. Phase-contrast images of bacterial migrations were recorded on microscopy using 100x objective.


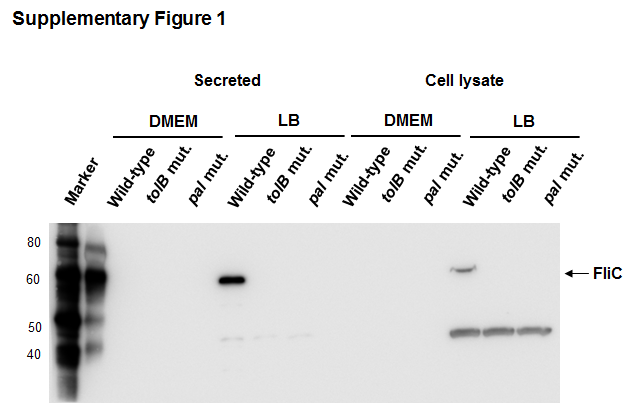


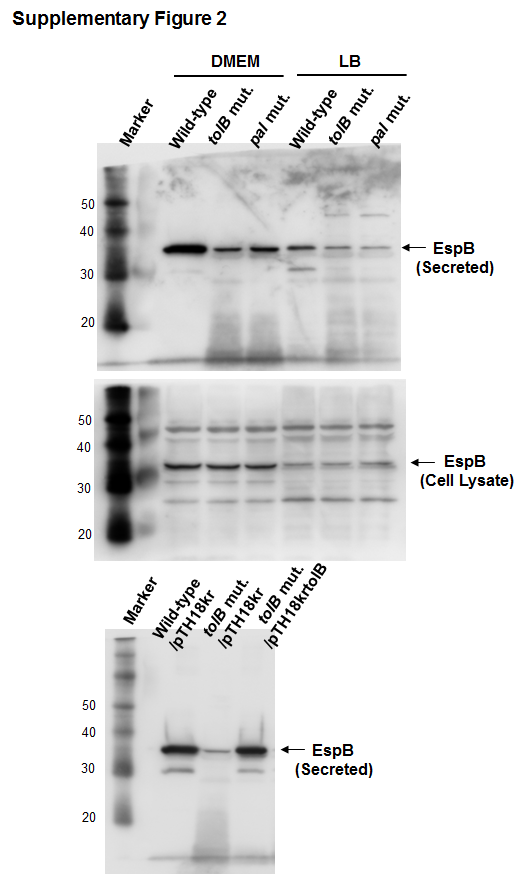


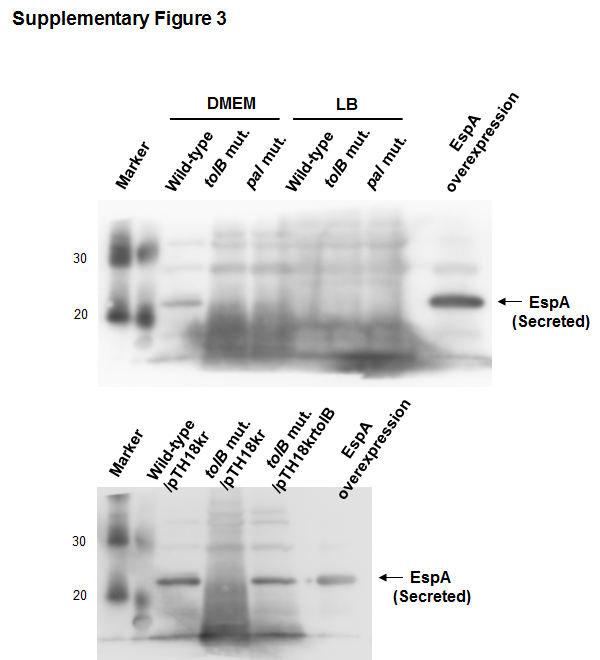

Supplement: Supplementary file 1 — Supplementary Figures. [file 41598_2020_72412_MOESM1_ESM.doc]
